# Exhaust emissions of gaseous and particle size-segregated water-soluble organic compounds from diesel-biodiesel blends

**DOI:** 10.1007/s11356-023-26819-3

**Published:** 2023-04-14

**Authors:** Margarita G. Evtyugina, Cátia Gonçalves, Célia Alves, Sérgio M. Corrêa, Luiz Carlos Daemme, Renato de Arruda Penteado Neto

**Affiliations:** 1 Department of Environment, Centre for Environmental and Marine Studies (CESAM), University of Aveiro, 3810-193 Aveiro, Portugal; 2 Faculty of Technology, Rio de Janeiro State University, Resende, RJ 27537-000 Brazil; 3 LACTEC – Technology Institute for Development, Curitiba, PR 80210-170 Brazil

**Keywords:** Exhaust emissions, Dynamometer, Emission factors, Water-soluble organic acids, Diesel/biodiesel blends

## Abstract

This study assessed the emissions of gaseous pollutants and particle size distributed water-soluble organics (WSO) from a diesel vehicle fuelled with ultralow sulphur diesel (B0) and 10 (B10), 20 (B20), and 30% (B30) biodiesel blends in a chassis dynamometer tested under transient mode. Particulate emission sampling was carried out in an ultraviolet (UV) test chamber using a 10-stage impactor. Samples were grouped into three size fractions and analysed by gas chromatography-mass spectrometry. Increasing the biofuel ratio up to 30% in the fuel reduced WSO emissions by 20.9% in comparison with conventional diesel. Organic acids accounted for 82–89% of WSO in all tested fuels. Dicarboxylic acids were the most abundant compound class, followed by hydroxy, aromatic, and linear alkanoic acids. Correlations between compounds demonstrated that adding biodiesel to diesel fuel reduces the emissions of nitrogen oxides (NO_x_), benzene, toluene, ethylbenzene and xylenes (BTEX), methane (CH_4_), total and nonmethane hydrocarbons (THC and NMHC), and dicarboxylic and hydroxy acids, but increases emissions of carbon dioxide (CO_2_) and alkanoic and aromatic acids. Emissions of dicarboxylic and hydroxy acids were strongly correlated with the biodiesel content. WSO emissions of coarse and fine (1.0–10 μm) particles decreased with the increasing biofuel content in fuel blend. The total share of ultrafine (0.18–1.0 μm) and nanoparticles (< 0.18 μm) increased in WSOs emissions from B20 and B30 blends, when compared with petrodiesel. The biodiesel content also affected the chemical profile of WSO size fractions.

**Supplementary Information:**

The online version contains supplementary material available at 10.1007/s11356-023-26819-3.

## Introduction

Biodiesel has received increasing attention worldwide as an alternative fuel in vehicle engines due to the scarcity of conventional fossil fuels, energy security concerns, and environmental issues. This interest results from its renewable origin, biodegradability, lower greenhouse gas (GHG) emissions, reduction in harmful exhaust emissions, low toxicity, and health concerns (Damanik et al. 2018; Živković and Veljković 2018; Verma et al. 2019).

Biodiesel consists of long-chain fatty acid alkyl esters and is typically produced from vegetable oils or animal fats through transesterification reaction of lipids with short-chain monohydric alcohol in the presence of alkali, acid, or enzyme catalysts (Mathew et al. 2021; Moser 2009). More than 350 feedstocks have been identified for production of biodiesel (Atabani et al., 2012), and among them, according to Souza et al. (2018), soybean, rapeseed, and palm oils are the main sources. Rapeseed, palm, soybean, and sunflower oils are the most used in the European Union (Bockey 2019). Soybean is the main raw material in the USA and the Brazilian biodiesel sector (da Silva et al. 2019; Hoekman 2009; Kumar et al. 2013; Meira et al. 2015).

Diesel engines are among the major sources of carbon monoxide (CO), nitrogen oxides (NO_x_), hydrocarbons (HC), and particulate matter (PM) (Ghazali et al. 2015 and references therein). When compared to standard diesel, the reduction in polycyclic aromatic hydrocarbons (PAHs) and near zero sulphur content, as well as oxygen enrichment and increase in cetane number in biodiesel, can have a positive effect on combustion characteristics and/or on engine exhaust emissions (Amaral et al. 2017; Ashraful et al. 2015; Ferreira et al. 2008; Tsai et al. 2010; Wang et al. 2016). Biodiesel, regardless of generation, used directly without modification or blended with conventional diesel in any proportions, benefits the physicochemical properties of the fuel and reduces exhaust emissions of diesel engines (Alptekin et al. 2015; Najafi 2018). Chemical compositions of biodiesel differ upon their origin and lead to variation in their properties and performance in terms of emission characteristics (Ghazali et al. 2015; Kumar et al. 2013). In general, most biodiesel blends result in a significant decrease in CO and total unburned HC emissions and significant increase in NO_x_ emissions (Ghazali et al., 2015; Hassan and Rahman 2017; Palani et al. 2022). Nevertheless, the use of biodiesel is not always favourable in terms of PM emissions, which may also depend on the refinement degree of fuel and after treatment technologies (Kontses et al. 2019; Wang et al. 2016 and references therein).

PM emissions have been always the main concern of manufactures due to their effect on the performance of the engines, environment, and human health. In fact, vehicular exhaust particles are related to adverse health effects such as airway inflammation (Ghio et al. 2012), vascular dysfunction (Mills et al. 2005), developmental toxicity (Ema et al. 2013), neuroinflammation (Levesque et al. 2011), and respiratory mortality (Atkinson et al. 2016), among others. Biological assays demonstrated that addition of biodiesel to diesel fuels can reduce PM emissions but not necessarily the adverse health outcomes (Fukagawa et al. 2013; Mehus 2015).

Particulate matter from vehicle exhausts contains a variety of chemical constituents such as elemental carbon (EC), organic carbon (OC), trace elements, metal oxides, a wide range of hydrocarbons (HC), organic oxygenated compounds, sulphur compounds, and other species (Cheung et al. 2010; Wang et al. 2016). These particles differ in size, composition, and solubility, which can directly influence their possible toxic properties. Its physical and chemical characteristics also depend on different parameters such as fuel properties, engine type, operating conditions, fuel injection mode, vehicle age and type, and after-treatment technology (Corrêa et al. 2021; Kontses et al., 2019; Verma et al. 2019).

Numerous studies have been focused on emissions from vehicles with traditional or alternative fuels. There are some recent literature reviews that summarised investigations on the performance and exhaust emission characteristics of biodiesel blends in diesel engines, including studies on the respective effects on human health (Damanik et al., 2018; Ghazali et al. 2015; Hasan and Rahman 2017; Palani et al. 2022; Verma et al. 2019). However, studies mentioned in these reviews discuss the impact of biodiesels and their blends on exhaust emissions of regulated pollutants (CO, HC, NO_x_, and PM) by environmental legislation in several countries. On the other hand, among unregulated pollutants, carbonyl compounds, BTEX (benzene, toluene, ethylbenzene, o-xylene, m-xylene, and p-xylene), EC, OC, total carbon (TC = EC + OC), and PAHs were the most characterised (Amaral et al. 2017; Bakeas and Karavalakis 2013; Bório et al. 2019; Borrás et al. 2009; Casal et al. 2014; Chiang et al. 2012; Corrêa et al. 2021; Corrêa and Arbilla 2006; Ferreira et al. 2008; Karavalakis et al. 2009, 2010a; Li et al. 2018; Lim et al. 2014; Martins et al. 2012; Wang et al. 2021). Cheung et al. (2010) conducted a more detailed study in a chassis dynamometer and determined emission factors (EFs) of particulate trace elements, metals, and solvent-extractable organic species (PAHs, hopanes, steranes, n-alkanes, and organic acids) from diesel or biodiesel passenger vehicles. Ghadikolaei et al. (2019) studied the PM chemical composition (TC, OC, EC, water-soluble organic carbon (WSOC), inorganic ions, metals, and elements) from a diesel engine fuelled with ternary fuel (diesel-biodiesel-ethanol) in different fuelling modes. However, few studies have investigated the chemical composition of biodiesel exhaust particulate matter in a size-segregated mode. Lin et al. (2008a, b) have assessed particle size distributions of PM and PAHs emitted from heavy-duty diesel engines fuelled with biodiesel blends. Rocha and Corrêa (2018) have characterised metals in coarse, micrometric, ultrafine, and nanoparticles in exhaust emissions from a typical diesel engine used by buses and trucks in Brazil fuelled with diesel-biodiesel blends. Size-segregated PAHs, nitro-PAHs, and alkyl-PAHs in emissions from diesel-biodiesel blends were studied by Corrêa et al. (2021). The volatile fraction (VOF) and the EC content in PM emissions were analysed according to different particle sizes, and the subsequent effect of oxygen content in biodiesel on the size-resolved characteristics of PM emissions was discussed by He et al. (2017). Recently, Li et al. (2021) measured the exhaust emissions of organic acids from gasoline, diesel, and liquefied petroleum gas (LPG) vehicles in China. The tests were performed in a dynamometer system using an iodide-adduct time-of-flight chemical ionisation mass spectrometer. Fuel-based and mileage-based emission factors of C_1_–C_5_ carboxylic acids, hydrogen cyanide, and isocyanic acid were obtained. It was subsequently concluded that emissions of carboxylic acids from diesel vehicles are much higher than those from gasoline vehicles.

WSOC can reach approximately 27–83% of the organic carbon mass of aerosols (Yu et al. 2004 and references therein) and is primarily emitted from combustion of biomass and fossil fuels, or secondarily formed from oxidation of VOCs, aromatic compounds, and high molecular weight hydrocarbons with anthropogenic or biogenic origin (Du et al., 2014; Park et al., 2015). WSOC affect the formation and physical properties of clouds, Earth’s radiative balance, and atmospheric chemistry with implications for regional and global climate change (Duarte et al., 2015, 2019; Niu et al. 2022; Tang et al., 2020). Moreover, due to their toxicity, particle-bound WSOC has been associated with human cardio-respiratory diseases (Cheung et al. 2009; Ramgolam et al. 2009). Water-soluble organic acids (WSOAs) are an important fraction of WSOC and have been comprehensively investigated across the world in different outdoor environments (Tang et al. 2020 and references therein). However, they have been sparsely documented in emissions from diesel/biodiesel combustion. Due to their high water solubility, carboxylic acids can potentially modify the hygroscopic properties of atmospheric particles, including their ambient size, and play an important role on cloud condensation nuclei activity (Kumar et al. 2003; Xu et al. 2010; Yu et al. 2000; Zhang et al. 2004). WSOAs are also relevant components of the secondary organic aerosol (SOA) and can contribute to the understanding of its chemical composition, sources, and formation mechanisms. Vehicle emissions were pointed out as an important source of WSOAs in urban environments (Bao and Sakamoto 2009; Bock et al. 2017; Kawamura et al. 1987, 2000; Li et al. 2021). However, dynamometer studies focused on the chemical composition of the water-soluble organic matter in exhaust emissions from engines fuelled with diesel, biodiesel, and biodiesel blends are, as far as we know, very scarce (Bock et al. 2017; Kawamura and Kaplan 1987; Kawamura et al., 2000). Also, most of these studies have assessed the concentrations of a limited number of organic water-soluble species in emissions mainly from older vehicle engines. Therefore, a more detailed characterisation of combustion emissions (e.g., emission factors) from diesel/biofuel engines is required for efficient and sustainable utilisation of biodiesel as an alternative to conventional fuel, as well as for source apportionment and for environmental control strategies. Moreover, the determination of the detailed molecular composition of WSOC has relevance for understanding the impacts of traffic emissions on physicochemical transformations in the atmosphere, ecosystems, and climate, as well as the associated effects on air quality and health. In this context, this paper presents results from a chassis dynamometer study conducted to determinate exhaust emissions from a diesel engine, using four binary mixtures of fossil diesel with biodiesel (0, 10, 15, 20, and 30%). It is important to underline that combustion conditions were kept the same between tests, which allowed evaluating the influence of the proportion of biodiesel in the fuel on the amount and chemical composition of exhaust engine emissions. The detailed particle size-segregated emission profiles of this study, with special focus on the water-soluble organic fraction, can be useful for source apportionment studies.

## Materials and methods

### Experimental facility

The tests were conducted with a chassis dynamometer belonging to the Lactec Laboratory, Institute of Technology for Development, Curitiba, Brazil. A light commercial vehicle Renault Master 2.5 L, 16 valve diesel engine with 84 kW at 3500 rpm, from 2012 with 71,000 km, was tested in the dynamometer system. A Horiba Mexa 7200 (CO, HC, NOx, CO_2_, CH_4_) bench analyser and 7500 DEGR (CO, HC, NOx, CO_2_, O_2_) were used to quantify the gaseous compounds. Total hydrocarbons (THC) were determined by flame ionisation detection (model FIA-720, 0–50 ppmC), carbon monoxide (CO) and carbon dioxide (CO_2_) were quantified by non-dispersive infrared spectrometry (model AIA-721A, 0–200 ppm, and AIA-722, 0–2.0% v/v, respectively), and nitrogen oxides (NOx) were measured by chemiluminescence (model CLA-720A, 0–50 ppm).

The vehicle engine was equipped with an after-treatment system that included a diesel oxidation catalyst (DOC) and an exhaust gas recirculation valve (EGR). This engine is a standard type widely used in the vehicle fleet of Brazilian towns and cities, such as school transport, company vans, and community transport. A constant volume sampler (CVS) was used to dilute the exhaust emissions with ambient air, which was pre-cleaned from particles through a quartz filter. Emission tests were performed in transient mode, according to the Brazilian standard ABNT NBR 6601 (2021), similar to the US FTP-75 standard, and included three phases (cold start transition phase, stabilised phase, and hot start transient phase).

The diluted emissions, after the measurement of the primary pollutants, were transferred to a reaction chamber, built with 5 mil Teflon FEP, inert to the exhaust gases and permeable to UV rays. The chamber had a volume of 3.9 m^3^, and included an aluminum support frame, resulting in dimensions of approximately 1.19 m × 1.19 m × 2.78 m. Both the sides and the bottom of the chamber were covered with a reflective shield to increase the incident radiation, as well as to prevent people from being exposed to UV rays. The simulation of sunlight was based on the work of Barnes and Rudzinki (2006), involving the use of 8 fluorescent lamps (Philips TUV30W G30T8 and UV–C and Bravo F30T8/BL UV-A) placed at the top of the appliance that emit UV radiation with peak at 254 nm and 365 nm, respectively. A 100-mm fan was placed inside the chamber (at the bottom) to achieve a better homogenisation of the gases.

### Fuels and sampling

The tested fuels included standard reference S10 (10 mg kg^−1^ of sulphur) pure diesel (B0) and four blends with soybean biodiesel B100 in the following proportions: B10, 10% volume of biodiesel, with a density of 835.0 kg m^−3^; B20, 20% biodiesel, with a density of 840.0 kg m^−3^; and B30, 30% biodiesel, with a density of 845.0 kg m^−3^.

Sampling of PM exhaust emissions was carried out using a 10-stage MSP nano MOUDI (micro-orifice uniform deposit impactor) II model 120R cascade impactor. In each stage, < 0.025-mm aluminium disks (MSP Corp. Subst Foil; 0100-96-0573A-X) with 47 mm diameter were used as substrates. The samples were collected from the Teflon fluorinated ethylene propylene (FEP) chamber after UV irradiance for 1 h. Accordingly, PM represents a mixture of non-reactive primary material emitted from the exhaust pipe and SOA formed in the reaction chamber. The sampling flow and the pressure drop were 30 L min^−1^ and 40 kPa, respectively. Particles were collected according to the following cut-point diameters: 10, 5.6, 3.2, 1.8, 1.0, 0.56, 0.32, 0.18, 0.10, and 0.056 μm. Samples were refrigerated (− 20 °C) immediately after the chassis dynamometer experiments.

An activated charcoal cartridge (Supelco ORBO 32 400/200 mg) was used for BTEX collection at a flow rate of 1.5 L min^−1^. The cartridges have 2 beds, a main one of 400 mg and a secondary one of 200 mg. Both beds were extracted, treated and chemically analysed in the same way. When the secondary bed has more than 5% of the total mass of analytes contained in the main bed, the sample is discarded, but this did not occur in any of the tests in this work. One cartridge was used for each phase of the standard protocol and another cartridge for the collection of dilution air. The contents of each cartridge were transferred to a 2-mL vial and added to 1000 μL of dichloromethane at − 20 °C. The flasks were capped with septum caps, placed in an ultrasonic bath for 20 min, and then allowed to rest for 1 h (Correa et al., 2012; Corrêa and Arbilla, 2007, 2006; Daemme, 2016a; Daemme, 2016b; Garcia et al., 2013; Macedo et al., 2017; Martins et al., 2016, 2007).

Carbonyl emissions (RCHO) were sampled using impingers with 2,4-dinitrophenylhydrazine acid solution (2,4-DNPH) following the guidelines of ABNT NBR 12026 (2021).

### Chemical analyses

The PM samples were grouped into three fractions to obtain enough mass for ulterior chemical analysis, as follows: coarse and fine fraction (1.0–10 μm), ultrafine particles (0.18–1.0 μm), and nanoparticles (< 0.18 μm).

Each filter set was ultrasonically extracted with 10 mL of ultra-pure water for 30 min with a 5-min stop in the middle. The extract was filtrated through a 13-mm PVDF syringe filter with 0.2-μm pore size (Whatman™, Buckinghamshire, United Kingdom) to remove any insoluble particles. The water extract was concentrated using a Turbo Vap® II concentrator (Biotage) and dried under a nitrogen stream.

Prior to speciation, oxygenated compounds were converted into the corresponding trimethylsilyl derivatives by addition of N,O-bis (trimethylsilyl) trifluoroacetamide (BSTFA):trimethylchlorosilane (TMCS) 99:1 (Supelco) and pyridine containing 2 internal standards: 1-chlorohexadecane (Merck) and tetracosane-d50 (Aldrich). The reaction mixture was heated in an oven at 70 °C for 3 h. The silylated derivatives were analysed by gas chromatography-mass spectrometry (GC-MS) from Thermo Scientific (Trace Ultra, quadrupole DSQ II) equipped with a split/splitless injector and a fused silica capillary column (TRB-5MS, 60 m × 0.25 mm × 0.25 μm). Helium was used as carrier gas at a constant flow of 1.2 mL min^−1^. The oven temperature program was as follows: 60 °C (1 min); 60–150 °C (10 °C min^−1^), 150–290 °C (5 °C min^−1^), and 290 °C (30 min). The derivatised extracts were analysed in both full scan and selected ion monitoring (SIM) modes. The acquisition mode was electronic impact at 70 eV, and the scanned masses ranged from 50 to 850 m/z. The GC–MS calibration was performed via injection of authentic standards in six concentration levels (5–50 ppm). Standards and samples were both co-injected with internal standards. All chemicals used were of analytical reagent grade from Sigma-Aldrich. The detection limit (LD) and quantification limit (LQ) for WSOAs varied from 0.03 to 0.70 and from 0.07 to 20.8 ppm, respectively, depending on the compound. Generally, the extraction recovery ranged between 77 and 99%. Extraction recovery tests were carried out with five blank filters, spiked with known quantities of the target analytes (standards). The impregnated filters were subsequently extracted and analysed following the procedures described above. The recovery was calculated as the ratio between the concentration of the standard determined after and before the extraction, expressed in percentage. EFs of the exhaust emission were expressed in a distance-based approach.

The BTEX chemical analyses were performed by GC-MS on a Varian 450GC 220MS chromatograph using a VF-5MS column (30 m, 0.25 mm, and 0.25 μm). Injections of 1.0 μL of sample were conducted at 200 °C, with a split ratio of 1:4, using helium 5.0 as carrier gas at 2.0 mL min^−1^. The initial column temperature was 40 °C, which was maintained for 3 min, and then followed by a heating rate of 15 °C min^−1^ up to 200 °C, which was held for 6 min. The temperatures of the ion trap, manifold, and transfer line were 150 °C, 40 °C, and 180 °C, respectively. The MS detector monitored ions from 72 to 79, 89 to 93, 101 to 107, and 119 to 121 (m/z) (Correa et al., 2012; Corrêa and Arbilla, 2007, 2006; Daemme, 2016a; Daemme, 2016b; Garcia et al., 2013; Macedo et al., 2017; Martins et al., 2016, 2007). The calibration was performed with a standard BTEX solution (Supelco EPA TO-1 Mix 1A) by external standardisation with concentrations of 0.10, 0.20, 0.50, 1.00, 2.00, and 4.00 ng mL^−1^, as an acceptance criterion of the analytical curve determination coefficients higher than 0.99. The calculated quantification limit for each BTEX compound was 5.6 pg mL^−1^, which corresponds to a concentration of 1.0 mg m^-3^ in the gas phase (Correa et al. 2012). All the measurements were within the analytical curves for all the samples and no dilution was necessary.

Carbonyl chemical analyses were performed by high performance liquid chromatography (HPLC) on an Agilent LC1200 with a G1314D detector at 365 nm. A volume of 20 μL was injected using a ZORBAX ODS column (25 cm × 4.6 mm × 5.0 μm) maintained at 35 °C. The mobile phase employed was 65% acetonitrile and 35% water at a constant flow of 1.0 mL min^−1^. The analytical curves were prepared by means of two types of standards, a Supelco 47650-U Mix and CRM47651 Mix.

### Data treatment

R language version 3.3.1 (Core Team 2016) was used for the processing of the study data. Correlations were calculated with 95% significance.

## Results and discussion

### Exhaust emissions of primary regulated and unregulated pollutants

Addition of biodiesel to conventional diesel led to significant reductions in emissions of BTEX, THC, and nonmethane hydrocarbons (NMHC) (Table 1). The high oxygen content in biodiesel promotes a more complete combustion, which reduces unburned hydrocarbon emissions (Ghazali et al., 2015; Hassan and Rahman 2017). Regarding regulated pollutants, emission levels of CO and NOx, for all tested fuels, were greatly above the Euro 5 and Euro 6 emission limits. PM emissions also exceeded both limits (Table 1).

**Table 1:** Emission factors (g km^−1^) of primary emissions of regulated and unregulated pollutants for each fuel type and emissions limits (g km^−1^) for diesel light commercial vehicles

Pollutant	Fuel type				Emission limits (g km^−1^)	
	B0	B10	B20	B30	EURO 5	EURO 6
NO_x_	1.51 ± 0.017	1.49 ± 0.043	1.44 ± 0.034	1.47 ± 0.021	0.18	0.08
CO	2.36 ± 0.050	2.32 ± 0.091	2.40 ± 0.080	2.29 ± 0.082	0.50	0.50
NMHC	0.438 ± 0.027	0.411 ± 0.011	0.375 ± 0.019	0.321 ± 0.024		
PM	0.061 ± 0.001	0.058 ± 0.055	0.063 ± 0.051	0.056 ± 0.045	0.005	0.0045
CO_2_	263 ± 2.07	262 ± 1.82	266 ± 2.22	267 ± 0.45		
CH_4_	0.012 ± 0.001	0.011 ± 0.001	0.011 ± 0.001	0.010 ± 0.001		
THC	0.447 ± 0.027	0.420 ± 0.011	0.384 ± 0.015	0.329 ± 0.023		
BTEX	0.0024 ± 0.001	0.0004 ± 0.001	0.0004 ± 0.001	0.0007 ± 0.001		
RCHO	0.103 ± 0.009	0.063 ± 0.013	0.095 ± 0.004	0.095 ± 0.007		

The effect of oxygenated fuel blends on the NO_x_ emission profiles have been investigated in numerous studies. However, the results from literature are complex and inconclusive. Many studies reported increased NO_x_ emissions with increasing biofuel content (Ghazali et al. 2015 and references therein). In contrast, a reduction in NO_x_ emissions with the use of biodiesel was observed in other studies (Abu-Hamdeh and Alnefaie 2015; Armas et al. 2010; Hoekman and Robbins 2012; Puhan et al. 2005; Serrano et al. 2015; Qi et al. 2009; Zhang et al., 2008). According to Ghazali et al. (2015), NO_x_ emissions are dependent on the fuel’s oxygen content. Higher oxygen contents and cetane number of biodiesel contribute to higher combustion temperature and therefore favour complete combustion and NO_x_ formation. Also, high NO_x_ emissions of biodiesel blends have been related to injection timing, driving cycle, and fuel proprieties (Karavalakis et al. 2010b; Lim et al. 2014). In this study, a slight decrease in NOx emissions was observed. This effect may be associated with the fact that the engine was equipped with an exhaust gas recirculation valve (EGR). The application of an EGR decriases the cylinder temperature (due to introduction of diluent gas of high specific heat) and reduces the oxygen content in the cylinder (Chuepeng et al. 2007; Zhang et al. 2008).

**Fig. 1:**
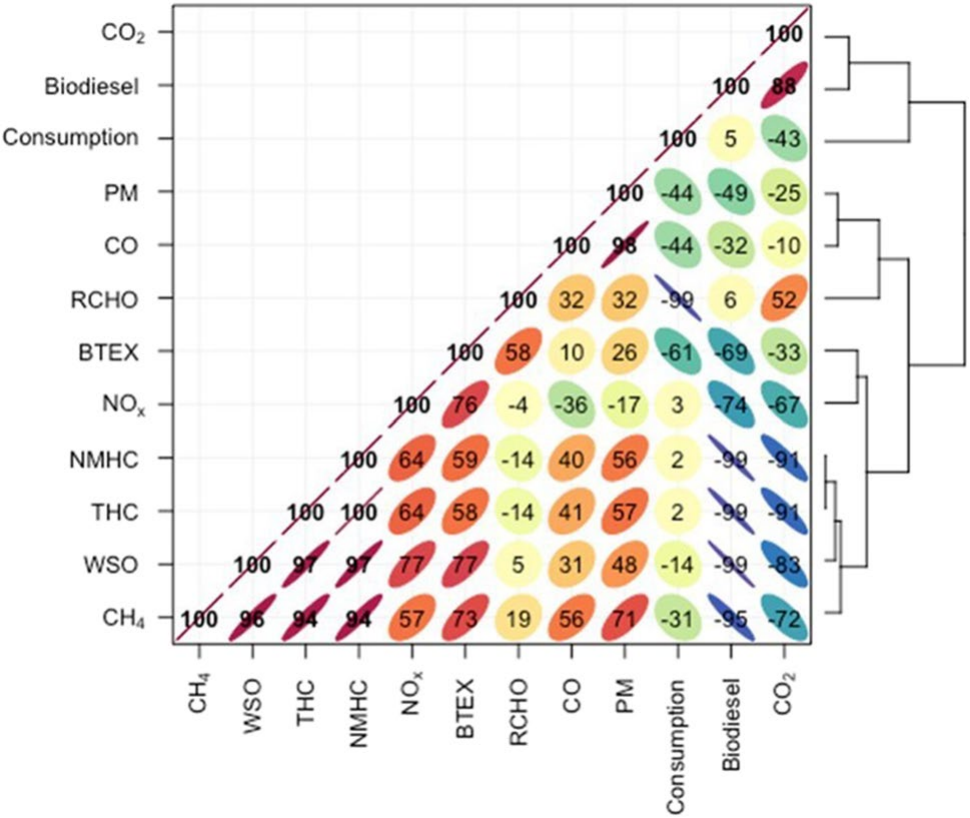
Pearson’s correlation matrix with dendrograms between biodiesel content, consumption, regulated/unregulated pollutants, and fraction of WSO

Figure 1 presents a Pearson’s correlation matrix between biodiesel content, consumption, regulated/unregulated pollutants, and the fraction of WSO. A cohesive data set was observed between the variables THC, NMHC, NO_x_, BTEX, and WSO, all with positive correlations (Fig. 1). Other strong positive correlations were registered between CH_4_ with WSO, THC, and NMHC. Also, CO_2_ presented very strong negative correlations with THC, NMHC, WSO, and CH_4_. The biodiesel content reduces the emissions of WSO, THC, NMHC, CH_4_, and BTEX to a greater degree, but indicates an increase in CO_2_. Fuel consumption was little influenced by biodiesel content but had a strong negative correlation with carbonyl compounds (RCHO).

### Exhaust emissions of water-soluble organics and size fractions

**Fig. 2:**
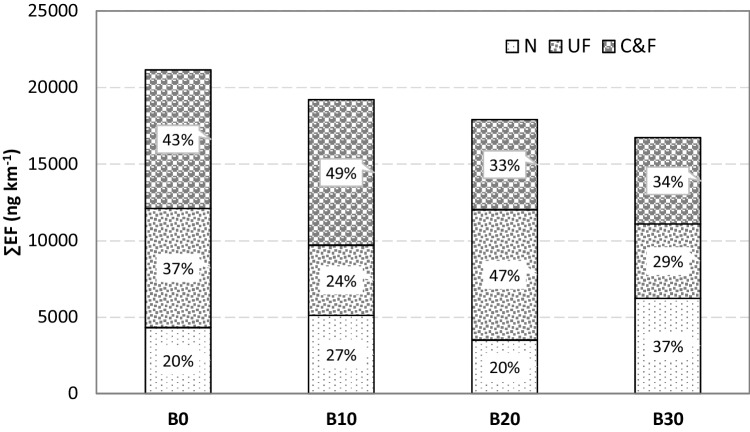
WSO emissions for different fuel blends divided into coarse and fine (C&F), ultrafine (UF), and nano (N) fractions

A decrease in emissions of WSO with increasing biofuel content in diesel was observed (Fig. 2). The reductions were 9.1, 14.9, and 20.9% for B10, B20, and B30 blends, respectively, when compared to diesel engine emissions. Considering that the operating conditions during chassis dynamometer testing were very uniform, this reduction can be related to the increase in the percentage of biofuel in the biodiesel blend. This assumption is confirmed by a very strong negative correlation between the biodiesel content and the emissions of WSO (Fig. 1).

Most constituents of fossil diesel are saturated hydrocarbons (primarily alkanes, including *n*, *iso*, and cycloalkanes). Aromatic compounds (naphthalenes and alkylbenzenes) are present in an even lesser amount (Al Qubeissi 2015). In the case of complete combustion, all organic matter of the fuel should be converted into CO_2_ and water. Alkanes are the most reduced compounds and, under combustion, are initially oxidised to alcohols, then to carbonyl compounds, then to carboxylic acids, then to esters, and finally to CO_2_ (Mkoma et al. 2014).

Soybean biodiesel mainly consists of methyl esters from linoleic, oleic, and palmitic acids, together with linolenic and stearic acids in lesser amount (Lam et al. 2010). Furthermore, the cetane number of biodiesels is higher than that of petrodiesel. Thus, the addition of biodiesel to fossil diesel decreases the share of saturated hydrocarbons and increases the cetane number and oxygen content in the fuel. According to Wang et al. (2021), the high cetane number and oxygen content of biodiesel contribute to a more complete combustion in the diesel engine and could explain the decrease of WSO emissions detected in the present study.

In the literature, there is no information on WSO emissions from biodiesel combustion. Scarce information was given about WSOC emissions. Cheung et al. (2009) conducted emission tests in a dynamometer facility for light-duty vehicles operated with petrodiesel (with 10 or 50 ppm of sulphur) and neat soybean biodiesel. Significant high WSOC emissions of 0.91, 1.48, and 1.42 mg km^−1^ were measured for a Honda Accord (Euro 4, diesel S10, capacity of 2.2 L), VW Golf TDi (Euro 2, 100% soybean biodiesel, capacity of 1.9 L), and VW Golf TDi (Euro 1, diesel S50, capacity of 1.9 L), respectively. A substantially lower emission (0.10 mg km^−1^) of WSOC was reported for the Honda Accord (Euro 4+) equipped with diesel particulate filter (DPF). The authors mentioned that to convert WSOC to water-soluble organic matter (WSOM), a factor of 2 can be used. Ghadikolaei et al. (2019) have investigated the effects of different fuelling modes on the composition of PM emissions under different operating conditions. An increase in WSOC, metals, and elements was observed, but a reduction in TC, EC, OC, and water-soluble inorganic carbon (WSIC) for blended fuel, when compared with diesel, was simultaneously registered. Worth noting that the amount, physical proprieties, and chemical composition of exhaust emissions vary greatly depending on different factors such as type of engine, fuel, operation conditions, and various technologies applied on the diesel engines and driving cycles, among others. So, the comparison of emissions from different studies is only adequate to indicate possible trends, main differences, and order of magnitude of emissions. Regarding the size profile, the share of coarse and fine particles (C&F) in WSO emissions decreased with increasing percentages of biofuel in diesel blends.

### Chemical composition of WSO emissions

**Fig. 3:**
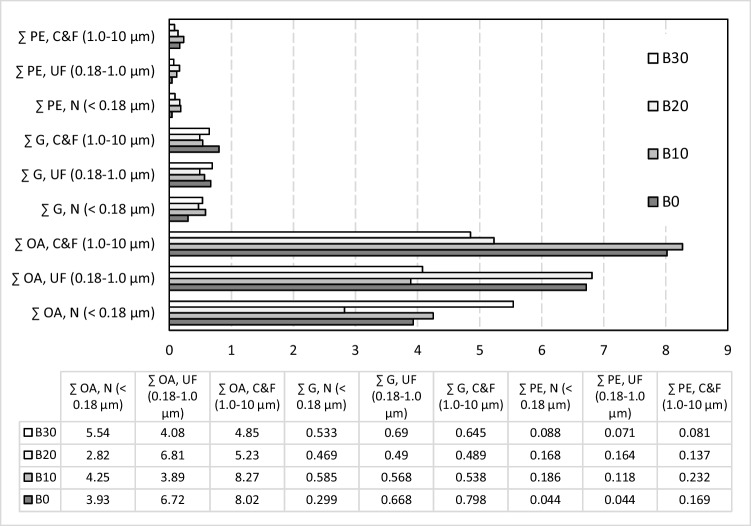
Emission factors (μg km^−1^) of identified classes and their contribution (%) to WSO emissions for each fuel type and size fraction (OA. organic acids; G, glycol; PE, polyethylene glycols)

Significant differences were also observed in the chemical composition of emissions according to size distribution. As it turned out in this study, the increase of biofuel content in diesel led to a decrease of WSO emissions. Organic acids were the major compound class, accounting for 82–89% of WSO. Following a similar behaviour of WSO, their ΣEFs decreased from 18.8 (B0) to 14.6 (B30) μg km^−1^ with the increase of biofuel content in diesel (Fig. 3).

Rather variable emissions of organic acids were observed in organic extracts from dynamometer tests by Cheung et al. (2010). In their study, ∑EFs of organic acids (C_9_–C_29_) were 1.04 ± 3.74 μg km^−1^, 342 ± 43 μg km^−1^, and 5240 ± 355 μg km^−1^ for diesel S10 (Euro 4, 2.2 L engine), 100% soybean biodiesel (Euro 2, 1.9 L), and diesel S50 (Euro 1, 1.9 L) vehicle engines, respectively. In the present study, significantly lower emissions were observed for glycerol (1.45 – 1.87 μg km^-1^) and polyethylene glycols (0.179 – 0.535 μg km^-1^) with no trend towards the percentage of biodiesel in fuels. No clear relationship was observed between the size distributed organic acids and biofuel content (Fig. 3). However, increased emissions of organic acids and glycerol with increased particle size were registered for the combustion of standard diesel (B0). Coarse and fine emissions of acids were *ca.* two times higher when compared with nanoparticles for B0, B10, and B20 blends. Nevertheless, the size distribution of emissions from B30 was fairly homogeneous. Ultrafine and coarse and fine fractions presented comparable acid emissions for B20, while the B10 blend had similar values for ultrafine and nanoparticles.

**Fig. 4:**
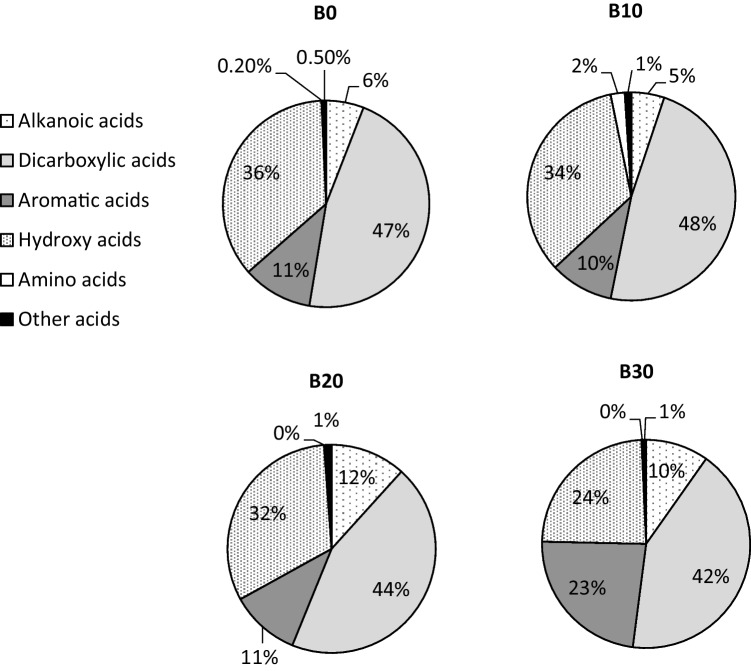
Share of emissions for different classes of organic acids and types of fuels

Six classes of organic acids were determined in WSO emissions (Fig. 4). Carboxylic acids, including alkanoic, dicarboxylic, aromatic, and hydroxy acids, were the most abundant. Their emission profile was very similar for B0, B10, and B20. However, in the case of the B30 experiment, aromatic acids practically doubled their share for WSO exhaust emissions (Fig. 4). Emissions of hydroxy acids were reduced from 32–36% to 24%, respectively. These results suggest that the addition of 30% biodiesel significantly affected the chemical composition of organic acid classes, while no notable changes were registered for other fuels tested.

Previous studies have pointed out that organic acids can be directly emitted from diesel engines as a result of combustion processes of the fuel (Bock et al. 2017; Kawamura et al. 2000). It was also suggested that C_1_–C_10_ organic acids can be formed in the atmosphere by photochemical oxidation of unsaturated hydrocarbons and aldehydes, which are emitted from motor exhaust in amounts of several orders of magnitude higher than organic acids (Kawamura et al. 2000).

**Fig. 5:**
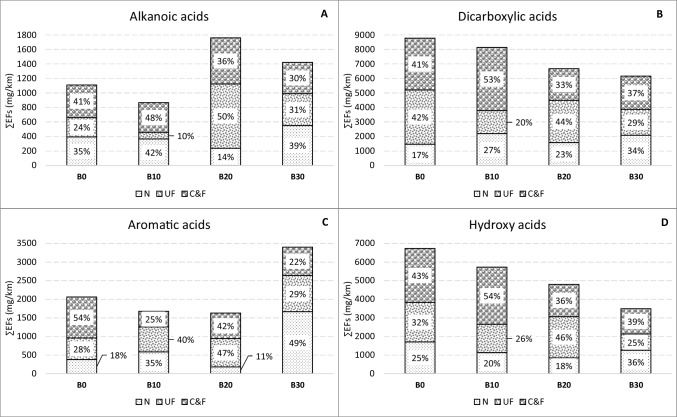
Carboxylic acid emissions of different classes in the coarse and fine (C&F), ultrafine (UF), and nano (N) fractions for each type of fuels

**Fig. 6:**
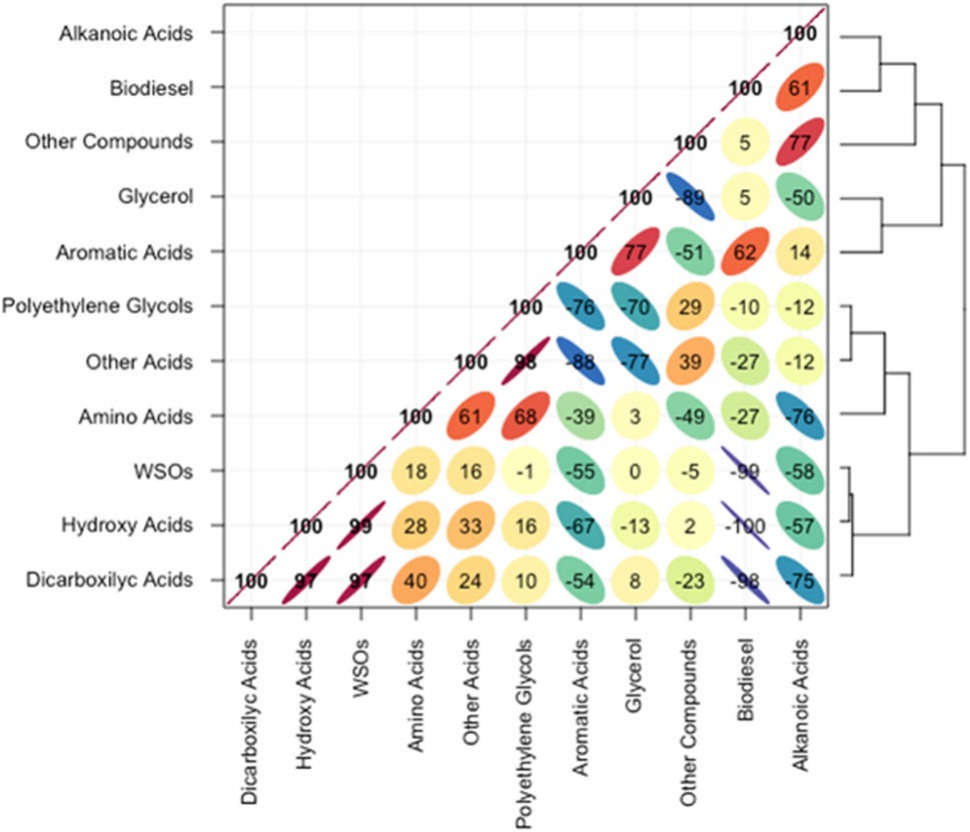
Pearson’s correlation matrix with dendrograms between biodiesel content and identified classes of organic compounds and WSO

Size-segregated exhaust emissions of different classes of carboxylic acids are shown in Fig. 5. Dicarboxylic and hydroxy acids were the most abundant with ∑EFs in the ranges 8.78–6.17 and 6.72–3.49 μg km^−1^, respectively. Their emissions were significantly decreased with rising biodiesel content in the blends. The reduction in emissions was 30% for dicarboxylic acids and 48% for hydroxy acids when comparing conventional diesel with the B30 biodiesel blend. Dicarboxylic acids were very strongly negatively correlated with biodiesel content in the fuel and positively correlated with hydroxy acids and with WSO (Fig. 6).

Likewise, hydroxy acids presented very strong negative correlations with biofuel content and positive correlations with WSO. This suggests that addition of biofuel in biodiesel blends leads to significant decreases in exhaust emissions of diacids and hydroxy acids, which possibly originate via similar pathways during the combustion process. Aromatic acids showed a different pattern (Fig. 5). Their ∑EFs from engine fuelled with B0, B10, and B20 were comparable and ranged between 1.63 and 2.06 μg km^−1^. However, an increase in emissions (3.39 μg km^−1^) was observed for B30.

Also, aromatic acids exhibited strong negative correlation with other acids, strong negative correlation with polyethylene glycols, moderate negative correlations with hydroxy acids, and strong positive correlation with glycerol (Fig. 6). Emissions of alkanoic acids from B0 and B10 tests were lower (0.87–1.11 μg km^−1^) when compared with B20 and B30 (1.42–1.76 μg km^−1^). Moderate correlations were observed for alkanoic acids and dicarboxylic acids, amino acids, hydroxy acids, glycerol, and other compounds (Fig. 6).

Regarding the size distribution, a percentage reduction in emissions of dicarboxylic acids, hydroxy acids, and alkanoic acids in coarse and fine particles *versus* ultrafine and nanoparticles was registered (Fig. 5). Aromatic acids presented an irregular size distribution profile, registering significant increases, up to 49%, in emissions of nanometric particles, from B30 *versus* 18% from B0.

### Emission factors of carboxylic acids

The individual size-segregated emission factors for dicarboxylic, aromatic, and hydroxy acids are presented in Table 2. Emissions of alkanoic acids and other compound classes, which had a minor contribution to WSO, are given in the Supporting Information (Table S5). The molecular structures of compounds listed in Tables 2 and S5 are presented in Figs. S1A, S1B, S1C, and S1D in the Supporting Information. Among dicarboxylic acids, oxalic and succinic acids were the most abundant in emissions of all tested fuels, whose EFs (sum of all size fractions) ranged between 3.79–6.25 and 1.04–2.04 μg km^−1^, respectively (Fig. S2). Regarding the size distribution, no trends were observed for emissions of oxalic acid. However, size-segregated EFs of other acids were generally higher for nanometric particles than for other size fractions (Table 2). Analysing the correlations between dicarboxylic acids (Table S1), it was observed a very strong positive relationship between malic-pimelic (*r* = 0.92), malic-thapsic (*r* = 0.90), pimelic-suberic (*r* = 0.90), pimelic-thapsic (*r* = 0.99), and suberic-azelaic (*r* = 0.92) acids. Also, oxalic-adipic (*r* = 0.75), succinic-glutaric (*r* = 0.70), malic-suberic (*r* = 0.83), malic-azelaic (*r* = 0.84), adipic-azelaic (*r* = 0.77), pimelic-azelaic (*r* = 0.75), and suberic-thapsic (*r* = 0.84) acids were strongly positively correlated. Dicarboxylic acids are highly oxidised compounds and usually show high water solubility. These organic acids extensively participate in various chemical and physical processes in the atmosphere and appear as a relevant part of WSOAs. Diacids mostly derive from secondary production (Kawamura and Yasui, 2005). However, Kawamura and Kaplan (1987) and Bock et al. (2017) argued that diesel engines are the primary sources of both gas and particle phase dicarboxylic acids. Kawamura and Kaplan (1987) analysed gas and particle phase concentrations of C_2_–C_10_ dicarboxylic acids emitted from a diesel engine without after-treatment system. In their study, oxalic, maleic, and methylmaleic diacids were detected as major species in exhaust emissions. It was suggested that incomplete combustion of cyclic olefines probably produces saturated diacids, which may be further oxidised to oxalic acid during combustion in motor exhausts. Azelaic acid is mostly formed by the photooxidation of biogenic unsaturated aliphatic acids (Kawamura and Kaplan 1987). However, this diacid was also detected in exhaust emissions by Kawamura and Kaplan (1987). The authors proposed that normal mono- and dicarboxylic acids can be the combustion products of normal alkanes in fuels. In other investigation, vapour-phase, semi-volatile, and particle-phase organic compounds from motor vehicles have been studied in a roadway tunnel (Fraser et al. 1998). Particulate emission rates of 97.3, 33.6, 7.5, and 9.5 μg L^−1^ fuel were reported for succinic, glutaric, adipic, and azelaic acids, respectively. In our study, the EFs for these four acids ranged between 0.580–1.14, 0.094–0.143, 0.073–0.118, and 0.076–0.149 μg L^−1^ fuel, respectively. The significant difference in emissions observed between Fraser et al. (1998) and the present study can be related to several factors such as variations in test methodology (measurements in roadway tunnel *vs* dynamometer tests), motor characteristics (older vehicle fleet operated in roadway tunnel *vs* more modern car tested in our study), fuels (gasoline or diesel-powered vehicles in roadway tunnel *vs* diesel or biodiesel blend in our study), and analytical methods (organic solvent extractions *vs* water extraction), among others.

**Table 2:** Emission factors of carboxylic acids (ng km^−1^) in WSO

	B0			B10			B20			B30		
	N	UF	C&F	N	UF	C&F	N	UF	C&F	N	UF	C&F
Dicarboxylic acids												
Ethanedioic (oxalic acid anhydrous) acid	1699	4464	3866	2011	1944	2632	1874	2644	1639	2825	2813	2681
Butanedioic (succinic) acid	357	456	811	471	312	1246	248	980	815	384	250	410
Hydroxybutanedioic (malic) acid	20.3	28.5	82.2	21.3	22.3	594	33.7	117	76.8	85.9	34.3	94.4
1,5-Pentanedioic (glutaric) acid	41.9	38.8	124	55.2	37.2	141	50.9	119.3	88.0	71.3	61.2	81.4
Hexanedioic (adipic) acid	28.2	31.1	154	30.8	13.9	156	49.2	44.6	51.4	48.4	29.8	53.0
Heptanedioic (pimelic) acid	2.47	2.65	12.8	6.33	1.92	35.0	4.81	5.84	6.50	11.2	5.81	11.0
Octanedioic (suberic) acid	6.01	13.60	39.3	6.56	3.75	85.7	8.26	10.2	15.6	19.5	10.3	25.0
Nonanedioic (azelaic) acid	48.17	45.15	176	8.89	4.64	451	31.9	45.6	82.2	33.2	19.0	84.6
Decanedioic (sebacic) acid	1.13	1.23	9.87	1.76	0.68	20.0	3.08	2.38	3.79	24.0	12.5	32.8
Hexanedecanedioic (thapsic) acid	0.23	Nd	0.28	0.20	Nd	3.70	0.18	0.34	0.18	0.76	0.43	1.08
Aromatic carboxylic acids												
Benzoic acid	573	727	1390	630	1004	576	211	967	828	2770	1463	661
4-Hydroxybenzoic acid	2.89	3.64	4.35	3.61	3.19	3.74	0.655	1.83	1.64	2.26	2.03	1.79
Trans-cinnamic acid	0.144	0.509	0.894	1.56	0.51	14.7	0.537	1.29	1.23	1.18	0.386	1.62
3-Hydroxybenzoic acid	2.02	2.23	2.92	1.41	1.88	6.55	1.26	1.60	0.938	1.85	2.14	2.18
Phthalic acid	7.65	7.48	39.6	5.67	3.00	77.2	10.3	11.9	18.7	14.2	7.32	9.83
Vanillic acid	4.02	10.6	17.6	1.20	0.54	2.52	0.48	2.99	4.10	1.18	0.95	1.28
Terephthalic acid	44.8	14.9	30.1	4.07	2.33	15.2	3.36	2.37	5.17	3.33	2.31	4.31
Syringic acid	0.449	0.191	0.285	0.272	0.236	1.02	0.08	0.22	0.20	0.36	0.29	0.59
Hydroxy acids												
Glycolic acid	1163	2659	3546	1758	1813	2697	839	3006	1857	1708	1062	1888
Glyceric acid	4.76	15.6	28.1	30.7	19.7	113	18.4	97.1	64.4	31.4	10.3	30.5
3-Hydroxypropanoic (hydracrylic) acid	41.2	190	262	152	95.7	337	75.7	192	89.3	79.1	45.5	88.6
3-Hydroxybutanoic (hydroxybutyric) acid	44.4	49.2	99.1	111	87.5	119	55.9	255	80.1	78.6	60.2	104
3,4-Dihydroxybutanoic acid	Nd	5.12	12.4	23.6	7.15	533	6.21	16.1	Nd	12.1	1.98	33.6
2-Hydroxysebacic acid	0.87	Nd	Nd	Nd	Nd	12.9	Nd	Nd	Nd	1.36	Nd	3.13

*Nd*, not detected

Seven hydroxy acids were detected in the WSO exhaust emissions (Table 2). Glycolic (hydroxyacetic) acid was the dominant species, accounting for 95% and 71–84% of all detected hydroxy acids in emissions of petrodiesel and diesel blends, respectively. Its emissions (all size fractions) ranged between 2.91 and 5.97 μg km^-1^ and presented a decrease with the addition of biofuel (Fig. S3). 3-Hydroxypropanoic acid was the second major hydroxy acid (EF = 0.21–0.58 μg km^−1^). With the exception of B10, emissions of this hydroxy acid from biodiesel blends were lower than that from conventional diesel. A very strong negative relationship (*r* = − 0.95) was observed between glycolic acid and biodiesel content (Table S2). 3-Hydroxypropanoic (hydracrylic) and 3-hydroxybutanoic acids were other abundant hydroxy acids. EFs of 3-hydroxybutanoic acid were also strongly negatively correlated (*r* = − 0.85) with biodiesel content. Additionally, positive and very strong (*r* = 0.96) and moderate (*r* = 0.68) correlations were observed between 3-hydroxybutanoic-glyceric and 3-hydroxypropanoic-3,4-dihydroxybutanoic acids, respectively. Except for 2-hydroxysebacic acid, which was not present in all the samples, a similar size distribution pattern was recorded for emissions from B0, B10, and B30 blends. The size-segregated EFs of hydroxy acids in nanometric particles were higher than those in ultrafine and coarse and fine size fractions. Worth noting that, up to date, there was no evidence that hydroxy acids can be primarily emitted from combustion of petrofuel/biodiesel in vehicle engines. Souza et al. (1999) reported the presence of glycolic and hydroxybutyric acids in a highly polluted urban atmosphere. The authors proposed biogenic emissions as possible sources for glycolic acid but have not established the origins of hydroxybutyric acid.

Benzoic acid was dominant among aromatic acids with EFs (all size fractions) between 1.86 and 3.33 μg km^−1^ (Fig. S4). Phthalic and terephthalic acids exhibited significant emissions with EFs between 31.3–85.9 and 9.95–89.9 ng km^−1^, respectively, and were the second most abundant species. Apart from cinnamic and syringic, all other acids were correlated with biodiesel content. Strong or moderate negative correlations were observed between biodiesel percentage and terephthalic (*r* = − 0.85), 4-hydroxybenzoic (*r* = − 0.80), vanillic (*r* = − 0.78), phthalic (*r* = − 0.62), and 4-hydroxybenzoic (*r* = − 0.47) acids, which generally experienced a reduction in exhaust emissions with the increase of biodiesel content (Table S4). In contrast, benzoic acid presented a positive moderate relationship (*r* = 0.67), recording very high emissions during the combustion of the B30 blend, when compared with petrodiesel. Likewise, high correlations were also observed between some of the aromatic acids. Terephthalic and vanillic acids showed very strong positive correlation (*r* = 0.98). 4-Hydoxybenzoic acid was strongly correlated with 3-hydroxybenzoic (*r* = 0.86) and phthalic (*r* = 0.74) acids. Strong positive correlations were observed between 3-hydroxybenzoic and phthalic (*r* = 0.84) and syringic (*r* = − 0.89) acids.

Emissions of aromatic acids, identified in the present work, did not show similar size-segregated distributions. EFs of benzoic and 4-hydroxybenzoic acids were higher for coarse and fine fraction and lower for nanoparticles during petrodiesel combustion (Table 2). However, exhaust emissions of these acids for the B30 blend were mostly found in nanoparticles, with the EFs for the coarse and fine size fraction being the lowest. An opposite pattern was observed for terephthalic and syringic acids, whose EFs were higher for nanoparticles from B0 and for the coarse and fine fraction from B30. It should be noted that an increase of biodiesel content led to an increase in emissions of ultrafine particles for benzoic acid and resulted in a reduction in emissions of coarse and fine particles of 4-hydroxybenzoic acid.

According to Kawamura et al. (1985, 2000) and Rogge et al. (1993), benzoic acid can be directly emitted from fossil fuel combustion. Phthalic and methylphthalic acids were documented among the major species in exhaust automobile emissions by Kawamura and Kaplan (1987). The authors proposed possible pathways for the formation of these acids from incomplete combustion of aromatic hydrocarbons (benzene, toluene, naphthalenes, and others) in car engines. Fraser et al. (1998) also found significant amounts of phthalic and terephthalic acids in emissions from motor vehicles in a roadway tunnel.

Homologous series of fatty C_8_–C_22_ straight-chain saturated monocarboxylic acids (alkanoic acids) were identified in the exhaust emissions (Fig. S5, Table S5). Among them, hexadecanoic acid was the most abundant compound in emissions from B0 and B10, followed by nonanoic and octanoic acids. In emissions from B20 and B30 blends, nonanoic acid was the dominant species, followed by hexadecenoic and octanoic acids. The highest EFs of all detected acids were registered for B20 or B30 blends, with the exception of C_16_ and C_18_ homologs, whose emissions were higher during the combustion of petrodiesel. Emissions of C_8_–C_15_ acids had very strong or strong correlations (*r* = 0.91–0.68) with biodiesel content (Table S5). Strong and moderate negative relationships with biodiesel content were recorded for emissions of hexadecanoic (*r* = 0.78) and octadecanoic (*r* = − 0.44) acids. Octanoic, nonanoic, decanoic, undecanoic, and dodecanoic acids showed very strong positive correlations (*r* = 0.97–1.0) among them, possibly indicating similar formation mechanisms of these compounds. Very strong negative correlations (*r* = − (0.93–0.99)) were recorded between C_13_ *vs* C_16_, C_18_ *vs* C_9_, C_18_ *vs* C_10_, C_18_ *vs* C_12_, and C_19_ *vs* C_17_ homologs. This suggests that the heavier acids could have decomposed, giving rise to lower molecular weight homologs. Likewise, strong positive or negative relationships were recorded for many other alkanoic acids (Table S5). According to Kawamura and Kaplan (1987), *n*-monocarboxylic acids can be combustion products of normal alkanes in fuels.

Regarding particle size, most of the individual EFs of alkanoic acids identified in B0 and B10 tests presented the highest value for coarse and fine and the lowest for fine fractions, respectively (Table S5). The exceptions were the hexadecanoic and octadecanoic acids, whose emissions were dominant in nanoparticles. However, in exhaust emissions from B20 and B30 blends, it was possible to observe a decrease in EFs of these acids in coarse and fine particles, while their EFs increased in ultrafine and nanoparticles for most of the homologs.

It is noteworthy that EFs of even chain fatty acids were significantly higher than emissions of odd chain fatty acids, except for C_8_ and C_9_ homologs. Cheung et al. (2010) investigated various classes of organic compounds from engine emissions fuelled with conventional diesel and 100% soybean biodiesel, reporting average EFs of C_8_–C_28_ alkanoic acids between 100 ng km^−1^ and 1000 μg km^−1^. In their study, similarly to ours, the emissions of even-chained acids were most abundant for C_12_–C_27_ homologs. Kawamura et al. (2000) documented concentrations of C_1_–C_9_ water-soluble monocarboxylic acids in gaseous and particulate phases of motor vehicle exhausts. In their study, in particulate emissions from Mercedes Benz 2200 (1971, Diesel, 2.2 l, 28000 miles), concentrations of monocarboxylic acids were dominated by odd chain homologs with an even/odd ratio of 0.68 for C_5_–C_10_ acids. Fraser et al. (1998) reported a very high emission of the order of 493.4 and 302.9 μg L^−1^ fuel for hexadecanoic (palmitic) and octadecanoic (stearic) acids, respectively. In the present study, EFs of C_16_ and C_18_ acids were significantly lower, ranging between 0.082–0.354 and 0.011–0.027 μg L^−1^ fuel, respectively.

Three nitro acids were detected in exhaust emissions (Table S5). Among them only pyroglutamic acid (5-oxo-L-proline) was present in emissions from all tested fuels, being the most abundant. Proline, glycine, and serine were found in soybean cultivars (Chavan et al. 2019; Qin et al. 2014). Also, some other water-soluble organic acids, including unsaturated and resin acids, were detected in exhaust emissions (Table S5). Generally, *cis*-pinonic (EFs = 14.6–64.8 ng km^−1^) and dehydroabietic (EFs = 10.3–75.9 ng km^−1^) acids were the dominant species. Pinic, citric, *cis*-9-octadecenoic (oleic), linoleic acid, dehydroabietic, and isopimaric acids were emitted during combustion of diesel and biodiesel blends in minor amounts.

Emissions of glycerol showed no significant changes with different types of fuel, ranging between 1.45 and 1.87 μg km^−1^ (all size fractions). However, its size distribution pattern changed with addition of biofuel. It was observed that in emissions from petrodiesel, the share in nanoparticles was about 17%, while the proportions in ultrafine and coarse and fine fractions were 38 and 45%, respectively. The particle size distribution of glycerol for different fuel blends was relatively homogeneous. Glycerol accounted for 28 to 37% of the total compound emissions for each size fraction. It can be present in biofuels as a contaminant (Bajpai and Tyagi 2006), which can explain its presence in exhaust emissions. Some other organic compounds, such as polyethylene glycols, urea, and fatty alcohols, were detected in exhaust emissions in insignificant amounts (Table S5). Some of these compounds are related to contaminations, lubricants, or ingredients in motor oil or catalytic additives.

## Conclusions

A study on the effects of different biodiesel-diesel blends on gaseous and particulate emissions from a diesel engine with the main focus on water-soluble organic fraction was carried out. Petrodiesel and B10, B20, and B30 blends were tested in a chassis dynamometer system under transient mode. The operation conditions were kept constant between tests, the only variable being the compositional differences of the fuel. Particulate size distributions of exhaust particles were also evaluated.

The results demonstrated that biodiesel blends affected the amounts, chemical composition, and size distribution pattern of exhaust emissions. It was observed that increasing the amount of biofuel up to 30% in the blends reduced WSO emissions by 20.8% in comparison with conventional diesel. Organic acids accounted for 82–89% of WSO in emissions from all tested fuels. Dicarboxylic acids were the most abundant compound class, followed by hydroxy, aromatic, and linear alkanoic acids. Emissions of dicarboxylic and hydroxy acids showed a reduction with increasing biofuel content. Aromatic and alkanoic acids were emitted in higher amounts from combustion of B30 and B20/B30 blends, respectively. Diacids and aromatic and alkanoic acids recorded very strong or moderate correlation with biodiesel content, indicating that these compounds can possibly have the same origins during the combustion process. Significant amounts of hydroxy acids were found in WSO exhaust emissions, also showing very strong correlations with the biodiesel content. Hydroxy acids are known to be derived from biological activities or generated from photochemical oxidation of biogenic or anthropogenic precursors. Aromatic acids accounted for 23% and about 11% of identified organic acid emissions from the combustion of the B30 blend and all other fuels tested, respectively. On the contrary, the lowest content of hydroxy acids in particulate emissions was recorded for the B30 blend.

The WSO content in coarse and fine particles decreased with the increase of biofuel content in the fuel blends. Although no emission pattern was registered for ultrafine and nanoparticles, an increase in the WSO content was observed in these two fractions of finer particles from B20 and B30 blends, when comparing with petrodiesel. The biodiesel content in the fuel also affected the chemical profile of particle size-segregated WSOs. Emission factors of about 50 water-soluble organic acids from diesel engine, fuelled with different biodiesel blends, were provided. The highest EFs were found for oxalic, glycolic, benzoic, and succinic acids. Glycerol and polyethylene glycol were also emitted in noteworthy amounts. Carboxylic acids represent a significant fraction of water-soluble carbon and play an important role on the CCN activity and PM growth. Correlations between pollutants demonstrated that adding biodiesel to diesel fuel reduces the emissions of NO_x_, BTEX, CH_4_, THC, NMHC, and dicarboxylic and hydroxy acids, but increases the emissions of CO_2_ and alkanoic and aromatic acids. The chemical speciation of water-soluble carboxylic acids in exhaust emissions constitutes a support tool in assessing the environmental impact of engine particulate emissions and in planning air quality control strategies. The use of biodiesel as blending compound for petrodiesel will increase in the coming decades due to several environmental, economic, and social advantages. Thus, the characterisation of the chemical composition and size distributions of engine-emitted particles will be of great interest in the future.

## Supplementary Information

ESM 1 The online version contains supplementary material available at XXXX (DOCX 529 KB).

## Data Availability

All data generated or analysed during this study are available from the corresponding author on reasonable request.
