# Supplementary material for: Exhaust emissions of gaseous and particle size-segregated water-soluble organic compounds from diesel-biodiesel blends
Source: Environ Sci Pollut Res Int. 2023 Apr 14;30(23):63738–53. doi: 10.1007/s11356-023-26819-3 (PMC10172243; doi:10.1007/s11356-023-26819-3)
Supplement: Supplementary file 1 — The online version contains supplementary material available at XXXX (DOCX 529 KB). [file 11356_2023_26819_MOESM1_ESM.docx]

**Determination of size-segregated water-soluble organic compounds in diesel-biodiesel blends exhaust emissions**

Margarita Evtyugina^1^, Cátia Gonçalves^1^, Sérgio Correia^2^, Célia Alves^1^, Luiz Carlos Daemme^3^, Renato de Arruda Penteado Neto^3^

^1^Department of Environment, Centre for Environmental and Marine Studies (CESAM), University of Aveiro, 3810-193 Aveiro, Portugal

^2^Rio de Janeiro State University, Faculty of Technology, Resende, RJ 27537-000, Brazil

^3^ LACTEC – Technology Institute for Development, Curitiba, PR, Brazil, 80210-170

**SUPLEMENTARY MATERIAL**

Table S1 Pearson’s correlation coefficients between biodiesel content (BD) and EFs of dicarboxylic acids

|  | *BD* | *Oxalic acid* | *Succinic acid* | *Malic acid* | *Glutaric acid* | *Adipic acid* | *Pimelic acid* | *Suberic acid* | *Azelaic acid* | *Sebacic acid* | *Thapsic acid* |
| --- | --- | --- | --- | --- | --- | --- | --- | --- | --- | --- | --- |
| *BD* | 1.00 |  |  |  |  |  |  |  |  |  |  |
| *Oxalic acid* | **-0.79** | 1.00 |  |  |  |  |  |  |  |  |  |
| *Succinic acid* | -0.47 | -0.16 | 1.00 |  |  |  |  |  |  |  |  |
| *Malic acid* | -0.09 | -0.34 | 0.48 | 1.00 |  |  |  |  |  |  |  |
| *Glutaric acid* | 0.28 | **-0.77** | **0.70** | 0.30 | 1.00 |  |  |  |  |  |  |
| *Adipic acid* | **-0.96** | **0.75** | 0.42 | 0.30 | -0.36 | 1.00 |  |  |  |  |  |
| *Pimelic acid* | 0.04 | -0.26 | 0.12 | **0.92** | -0.01 | 0.22 | 1.00 |  |  |  |  |
| *Suberic acid* | -0.37 | 0.17 | 0.18 | **0.83** | -0.26 | 0.61 | **0.90** | 1.00 |  |  |  |
| *Azelaic acid* | **-0.61** | 0.21 | 0.53 | **0.84** | -0.04 | **0.77** | **0.75** | **0.92** | 1.00 |  |  |
| *Sebacic acid* | **0.73** | -0.26 | **-0.87** | -0.07 | -0.41 | -0.59 | 0.27 | 0.04 | -0.36 | 1.00 |  |
| *Thapsic acid* | 0.17 | -0.35 | 0.05 | **0.90** | 0.01 | 0.09 | **0.99** | **0.84** | **0.66** | 0.37 | 1.00 |

Table S2 Pearson’s correlation coefficients between biodiesel content (BD) and EFs of hydroxy acids

|  | *BD* | *Glycolic acid* | *Glyceric acid* | *3-Hydroxy-propanoic acid* | *3-Hydroxy-butanoic acid* | *3,4-Di-*  *hydroxy-*  *butanoic acid* | *2-Hydroxy-sebacic acid* |
| --- | --- | --- | --- | --- | --- | --- | --- |
| *BD* | 1.00 |  |  |  |  |  |  |
| *Glycolic acid* | **-0.95** | 1.00 |  |  |  |  |  |
| *Glyceric acid* | 0.17 | -0.36 | 1.00 |  |  |  |  |
| *3-hydroxypropanoic acid* | **-0.85** | **0.64** | 0.25 | 1.00 |  |  |  |
| *3-Hydroxybutanoic acid* | 0.33 | -0.45 | **0.96** | 0.02 | 1.00 |  |  |
| *3,4-Dihydroxybutanoic acid* | -0.22 | -0.10 | 0.48 | **0.68** | 0.24 | 1.00 |  |
| *2-Hydroxysebacic acid* | -0.05 | -0.26 | 0.31 | 0.49 | 0.09 | **0.96** | 1.00 |

Table S3 Pearson’s correlation coefficients between biodiesel content (BD) and EFs of aromatic acids

|  | *BD* | *Benzoic acid* | *4-Hydroxy-benzoic acid* | *Cinnamic acid* | *3-Hydroxy-benzoic acid* | *Phthalic acid* | *Vanillic acid* | *Terephthalic acid* | *Syringic acid* |
| --- | --- | --- | --- | --- | --- | --- | --- | --- | --- |
| *BD* | 1.00 |  |  |  |  |  |  |  |  |
| *Benzoic acid* | **0.67** | 1.00 |  |  |  |  |  |  |  |
| *4-Hydroxybenzoic acid* | **-0.80** | -0.28 | 1.00 |  |  |  |  |  |  |
| *Cinnamic acid* | -0.16 | -0.38 | 0.44 | 1.00 |  |  |  |  |  |
| *3-Hydroxybenzoic acid* | **-0.47** | -0.16 | **0.86** | **0.78** | 1.00 |  |  |  |  |
| *Phthalic acid* | **-0.62** | -0.64 | **0.74** | **0.87** | **0.84** | 1.00 |  |  |  |
| *Vanillic acid* | **-0.78** | -0.24 | 0.53 | -0.47 | 0.03 | 0.02 | 1.00 |  |  |
| *Terephthalic acid* | **-0.85** | -0.24 | **0.69** | -0.30 | 0.23 | 0.18 | **0.98** | 1.00 |  |
| *Syringic acid* | -0.03 | 0.25 | 0.59 | 0.72 | **0.89** | 0.58 | -0.30 | -0.10 | 1.00 |

Table S4 Pearson’s correlation coefficients between biodiesel content (BD) and EFs of alkanoic acids

|  | *BD* | *C8* | *C9* | *C10* | *C11* | *C12* | *C13* | *C14* | *C15* | *C16* | *C17* | *C18* | *C19* | *C20* | *C22* |
| --- | --- | --- | --- | --- | --- | --- | --- | --- | --- | --- | --- | --- | --- | --- | --- |
| *BD* | 1.00 |  |  |  |  |  |  |  |  |  |  |  |  |  |  |
| *C8* | **0.83** | 1.00 |  |  |  |  |  |  |  |  |  |  |  |  |  |
| *C9* | **0.68** | **0.97** | 1.00 |  |  |  |  |  |  |  |  |  |  |  |  |
| *C10* | **0.79** | **1.00** | **0.98** | 1.00 |  |  |  |  |  |  |  |  |  |  |  |
| *C11* | **0.85** | **0.99** | **0.96** | **0.99** | 1.00 |  |  |  |  |  |  |  |  |  |  |
| *C12* | **0.72** | **0.97** | **1.00** | **0.98** | **0.97** | 1.00 |  |  |  |  |  |  |  |  |  |
| *C13* | **0.85** | **0.57** | 0.45 | 0.54 | **0.65** | 0.53 | 1.00 |  |  |  |  |  |  |  |  |
| *C14* | **0.91** | **0.78** | **0.62** | **0.74** | **0.76** | **0.61** | 0.56 | 1.00 |  |  |  |  |  |  |  |
| *C15* | **0.69** | 0.21 | -0.02 | 0.15 | 0.23 | 0.01 | 0.56 | **0.74** | 1.00 |  |  |  |  |  |  |
| *C16* | **-0.78** | -0.50 | -0.40 | -0.47 | -0.58 | -0.48 | **-0.99** | -0.45 | -0.49 | 1.00 |  |  |  |  |  |
| *C17* | 0.42 | -0.16 | -0.36 | -0.22 | -0.10 | -0.29 | **0.63** | 0.28 | **0.81** | **-0.63** | 1.00 |  |  |  |  |
| *C18* | -0.44 | **-0.87** | **-0.96** | **-0.90** | **-0.85** | **-0.94** | -0.22 | -0.40 | 0.30 | 0.18 | **0.60** | 1.00 |  |  |  |
| *C19* | -0.21 | 0.37 | 0.57 | 0.43 | 0.34 | 0.53 | -0.31 | -0.22 | **-0.82** | 0.30 | **-0.93** | **-0.78** | 1.00 |  |  |
| *C20* | -0.28 | **-0.67** | **-0.71** | **-0.68** | -0.59 | **-0.64** | 0.21 | -0.52 | 0.11 | -0.30 | **0.66** | **0.76** | -0.59 | 1.00 |  |
| *C22* | -0.01 | -0.47 | **-0.66** | -0.53 | -0.48 | **-0.66** | -0.07 | 0.19 | 0.72 | 0.11 | **0.67** | **0.80** | **-0.90** | 0.34 | 1.00 |

Table S5. Emission factors of WSO (ng/km)

|  | B0 | | | B10 | | | B20 | | | | | | B30 | | | | | |  |  |
| --- | --- | --- | --- | --- | --- | --- | --- | --- | --- | --- | --- | --- | --- | --- | --- | --- | --- | --- | --- | --- |
|  | N | UF | F | N | UF | F | N | | UF | | F | | N | | UF | | F | |  |  |
| ***Straight-chain, saturated carboxylic acids (alkanoic acids)*** | | | | | | | | | | | | | | | | | | |  |  |
| Octanoic (caprylic) acid | 22.9 | 5.34 | 67.0 | 30.4 | 3.80 | 59.4 | 25.4 | | 169 | | 106 | | 126 | | 68.5 | | 59.4 | |  |  |
| Nonanoic (pelargonic) acid | 43.8 | 3.34 | 131 | 56.4 | 1.70 | 125 | 87.3 | | 370 | | 220 | | 200 | | 127 | | 109 | |  |  |
| Decanoic (caproic) acid | 16.7 | 2.15 | 38.3 | 25.8 | 0.927 | 52.9 | 25.6 | | 67.0 | | 50.9 | | 52.4 | | 33.8 | | 31.6 | |  |  |
| Undecanoic acid | 2.50 | 0.466 | 5.01 | 2.39 | 0.836 | 4.42 | 4.72 | | 8.17 | | 7.05 | | 7.74 | | 4.57 | | 5.06 | |  |  |
| Dodecanoic (lauric) acid | 9.04 | 1.70 | 23.4 | 14.8 | 1.47 | 26.6 | 56.5 | | 28.7 | | 28.5 | | 36.9 | | 19.2 | | 22.1 | |  |  |
| Tridecanoic acid | 0.71 | 0.332 | 1.51 | 2.88 | 0.435 | 4.23 | 2.15 | | 2.44 | | 3.21 | | 3.52 | | 1.89 | | 3.85 | |  |  |
| Tetradecanoic (myristic) acid | 10.1 | 11.6 | 11.7 | 11.8 | 3.60 | 23.1 | 10.6 | | 9.30 | | 21.6 | | 13.8 | | 13.8 | | 25.3 | |  |  |
| Pentadecanoic acid | 1.95 | 1.95 | 2.69 | 3.87 | 1.54 | 2.31 | 1.62 | | 2.08 | | 1.22 | | 5.13 | | 6.14 | | 11.7 | |  |  |
| Hexadecanoic (palmitic) acid | 267 | 220 | 153 | 194 | 73.5 | 144 | 18.1 | | 222 | | 185 | | 89.6 | | 152 | | 142 | |  |  |
| Heptadecanoic acid | 0.533 | 0.434 | 0.608 | 3.20 | 0.290 | 1.33 | 0.332 | | 0.388 | | 0.278 | | 1.57 | | 1.67 | | 3.01 | |  |  |
| Octadecanoic (stearic) acid | 16.5 | 19.6 | 13.3 | 15.9 | 3.21 | 11.5 | 6.14 | | 5.16 | | 9.16 | | 12.7 | | 11.8 | | 19.1 | |  |  |
| Nonadecanoic acid | 0.551 | 0.920 | 0.330 | Nd | Nd | Nd | 0.642 | | 1.48 | | 1.03 | | 0.187 | | Nd | | 0.143 | |  |  |
| Eicosanoic (arachidic) acid | 0.327 | 0.230 | 0.385 | 0.708 | 0.065 | 0.50 | 0.178 | | 0.157 | | 0.130 | | 0.530 | | 0.459 | | Nd | |  |  |
| Docosanoic (behenic) acid | 0.162 | 0.046 | 0.422 | 0.550 | Nd | Nd | Nd | | Nd | | Nd | | Nd | | 0.301 | | 0.508 | |  |  |
| ***Amino acids*** |  |  |  |  |  |  |  | |  | |  | |  | |  | |  | |  |  |
| Glycine | Nd | Nd | Nd | 17.8 | Nd | 27.7 | Nd | | Nd | | Nd | | 9.55 | | Nd | | 10.1 | |  |  |
| L-Serine | Nd | Nd | Nd | Nd | Nd | Nd | Nd | | Nd | | Nd | | Nd | | Nd | | Nd | |  |  |
| 5-oxo-L-proline | Nd | 21.1 | 13.5 | 6.41 | Nd | 319 | Nd | | 15.6 | | Nd | | 8.021 | | 3.09 | | Nd | |  |  |
| ***Other acids*** | | | | | | | | | | | | | | | | | | | | |
| Cis-Pinonic acid | 9.91 | 5.46 | 23.0 | 22.7 | 5.36 | 12.7 | | 6.38 | | 17.62 | | 15.11 | | 6.61 | | 4.09 | | 3.87 | |  |
| Pinic acid | 2.67 | 2.02 | 8.67 | 3.74 | 1.60 | 4.44 | | 3.74 | | 5.38 | | 4.83 | | 3.52 | | 2.81 | | 3.95 | |  |
| Citric acid | 0.187 | 0.165 | 2.30 | 0.55 | 0.115 | 0.569 | | 0.392 | | 0.327 | | 0.842 | | 3.11 | | 0.748 | | 5.58 | |  |
| Cis,cis-9-12-octanedecanoic, 9-cis-hexadecenoic (linoleic) (C18:2)acid | 0.440 | 0.373 | Nd | 0.385 | 0.064 | Nd | | Nd | | Nd | | Nd | | Nd | | 0.173 | | Nd | |  |
| cis-9-Octadecenoic (oleic) acid | 1.01 | 1.15 | 0.565 | 1.70 | 0.168 | 1.52 | | 0.270 | | 0.461 | | 0.638 | | 0.334 | | 0.484 | | 0.778 | |  |
| Dehydroabietic acid | 7.72 | 3.89 | 3.65 | 4.30 | 3.14 | 5.07 | | 3.331 | | 60.8 | | 11.7 | | 2.43 | | 1.77 | | 6.08 | |  |
| Isopimaric acid | 9.92 | 9.00 | 9.30 | 9.46 | 10.7 | Nd | | 8.95 | | 9.23 | | 9.56 | | 8.92 | | 9.67 | | 8.27 | |  |
| ***Glycerol*** | 299 | 668 | 798 | 585 | 568 | 806 | | 469 | | 490 | | 489 | | 533 | | 690 | | 645 | |  |
| ***Polyethylene glycols*** |  |  |  |  |  |  | |  | |  | |  | |  | |  | |  | |  |
| Diethylene glycol | 16.5 | 27.4 | 70.8 | 98.8 | 74.0 | 77.1 | | 120 | | 126 | | 108 | | 48.9 | | 50.2 | | 44.4 | |  |
| Triethylene glycol | 26.3 | 16.3 | 96.2 | 85.1 | 42.7 | 42.1 | | 46.7 | | 37.1 | | 28.0 | | 37.0 | | 19.9 | | 35.3 | |  |
| Tetraethylene glycol | 1.25 | 0.405 | 1.76 | 1.64 | 1.32 | 0.649 | | 1.35 | | 0.968 | | 0.876 | | 1.94 | | 1.29 | | 1.44 | |  |
| 1-monostearin | 1.43 | 1.36 | 2.40 | 7.36 | Nd | Nd | | Nd | | Nd | | Nd | | Nd | | Nd | | Nd | |  |
| ***Other compounds*** | | | | | | | | | | | | | | | | | | | | |
| Urea | Nd | 288 | Nd | Nd | Nd | 52 | | Nd | | 998 | | Nd | | Nd | | Nd | | Nd | |  |
| Vanillin | 3.62 | 2.39 | 5.84 | 5.77 | 3.41 | 8.69 | | 8.55 | | 8.62 | | 5.04 | | 10.5 | | 8.10 | | 8.13 | |  |
| Hexadecanol | 2.91 | 2.87 | 3.40 | 2.34 | 4.56 | 3.55 | | 2.58 | | 2.66 | | 2.50 | | 3.33 | | 3.48 | | 3.79 | |  |
| Octadecanol | 1.07 | 2.11 | 1.25 | 2.05 | 1.24 | 4.13 | | 0.522 | | 0.695 | | 0.424 | | 1.44 | | 2.11 | | 2.08 | |  |


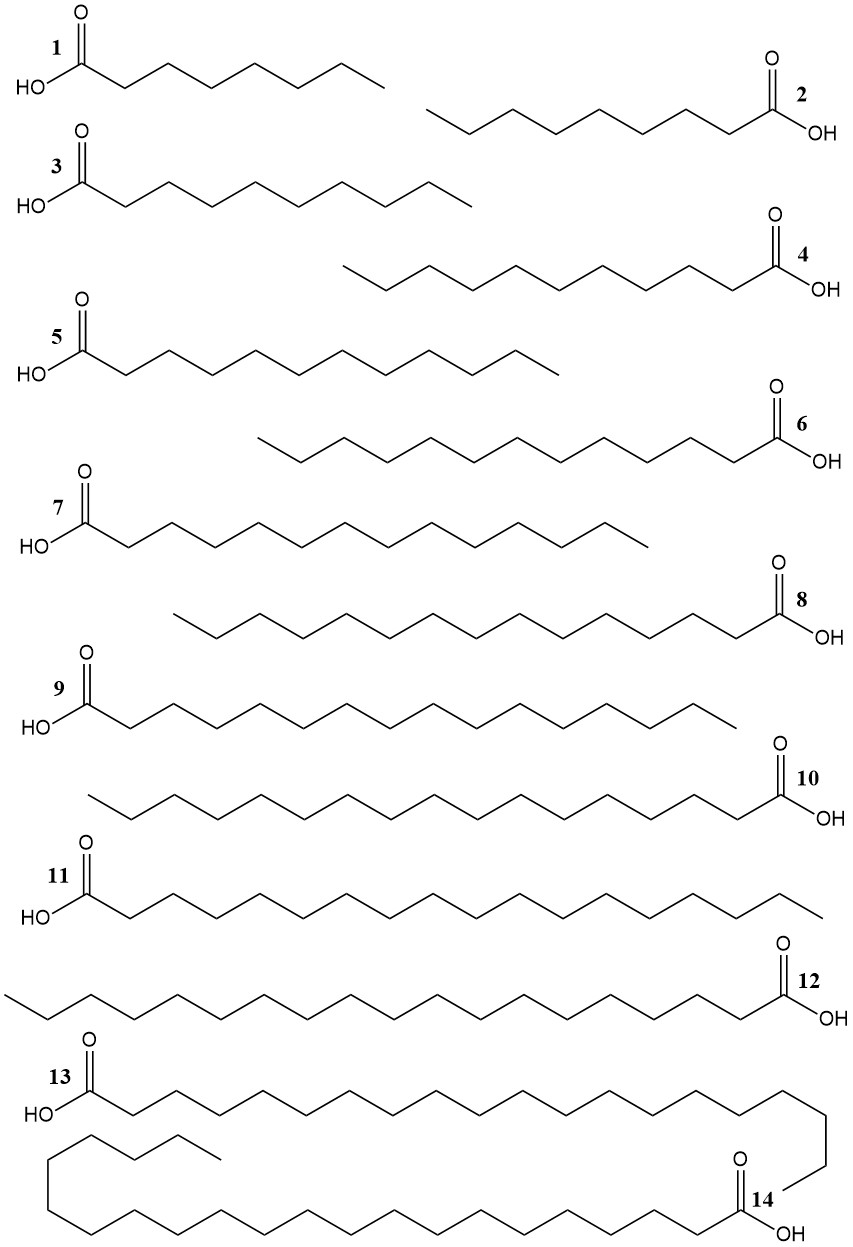


Figure S1A Molecular structure of alkanoic acids (1 – octanoic (caprylic) acid, 2 – nonanoic (pelargonic) acid, 3 – decanoic (caproic) acid, 4 – undecanoic acid, 5 – dodecanoic (lauric) acid, 6 – tridecanoic acid, 7 – tetradecanoic (myristic) acid, 8 – pentadecanoic acid, 9 – hexadecanoic (palmitic) acid, 10 – heptadecanoic acid, 11 – octadecanoic (stearic) acid, 12 – nonadecanoic acid, 13 – eicosanoic (arachidic) acid, 14 – docosanoic (behenic) acid


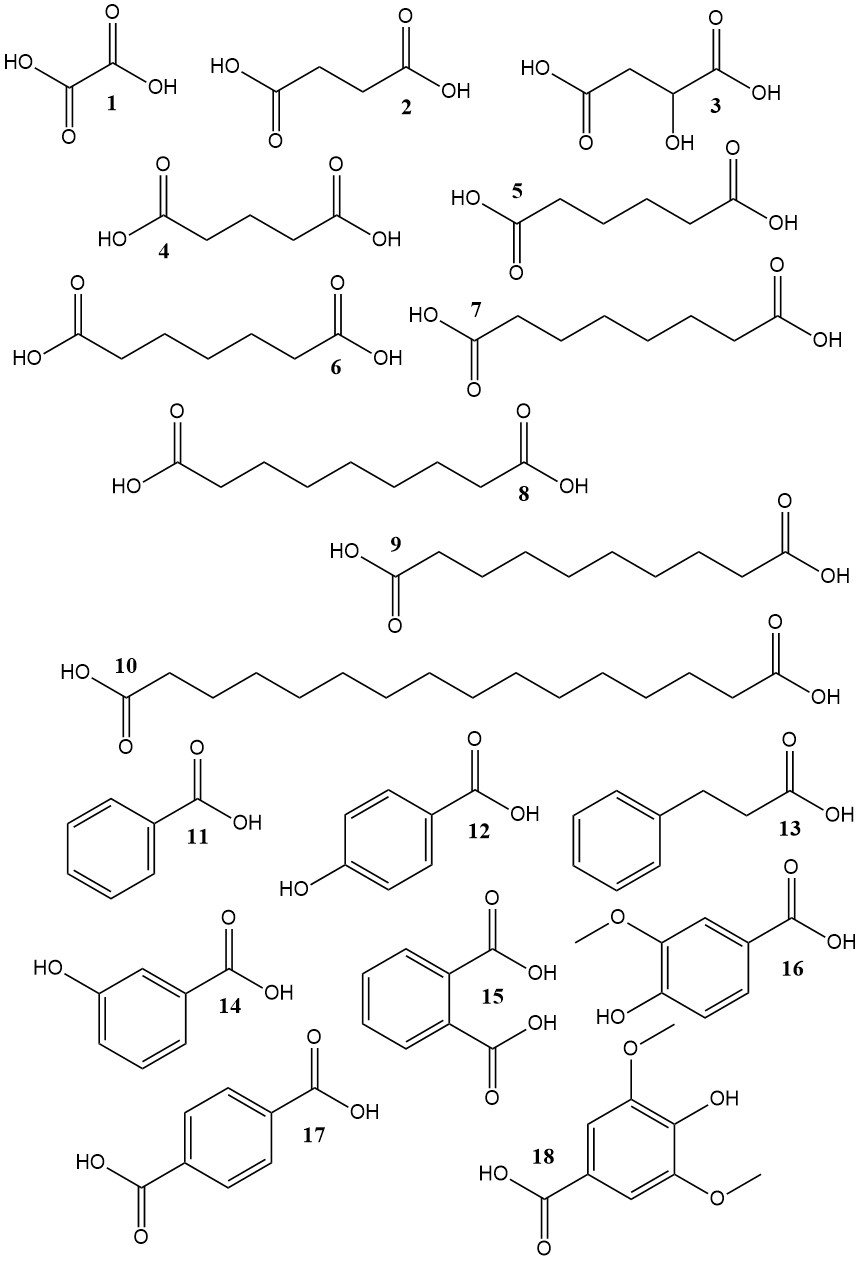


Figure S1B Molecular structure of dicarboxylic and aromatic acids (1 – ethanedioic (oxalic anhydrous) acid, 2 – butanedioic (succinic) acid, 3 – hydroxybutanedioic (malic) acid, 4 – 1,5-pentanedioic (glutaric) acid, 5 – hexanedioic (adipic) acid, 6 – heptanedioic (pimelic) acid, 7 – octanedioic (suberic) acid, 8 – nonanedioic (azelaic) acid, 9 – decanedioic (sebacic) acid, 10 – hexanedecanedioic (thapsic) acid, 11 – benzoic acid, 12 – 4-hydroxybenzoic acid, 13 – trans-cinnamic acid, 14 – 3-hydroxybenzoic acid, 15 – phthalic acid, 16 – vanillic acid, 17 – terephthalic acid, 18 – syringic acid


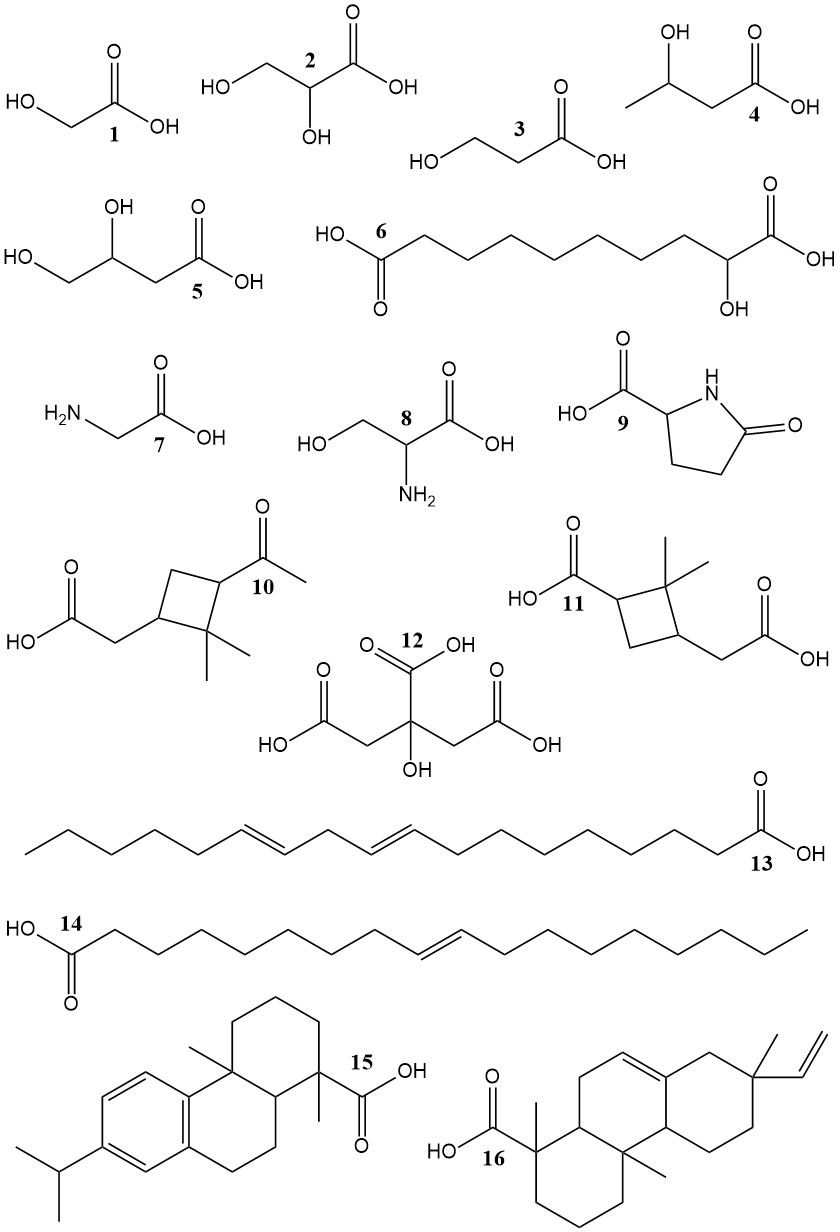


Figure S1C Molecular structure of hydroxy, amino- and other acids (1 – glycolic acid, 2 – glyceric acid, 3 – 3-hydroxypropanoic (hydracrylic) acid, 4 – 3-hydroxybutanoic (hydroxybutyric) acid, 5 – 3,4- dihydroxybutanoic acid, 6 – 2-hydroxysebacic acid, 7 – glycine, 8 – L-serine, 9 – 5-oxo-L-proline, 10 – cis-pinonic acid, 11 – pinic acid, 12 – citric acid, 13 – cis,cis-9-12-octanedecanoic, 9–cis-hexadecenoic (linoleic) (C18:2)acid, 14 – cis-9-octadecenoic (oleic) acid, 15 – dehydroabietic acid, 16 – isopimaric acid


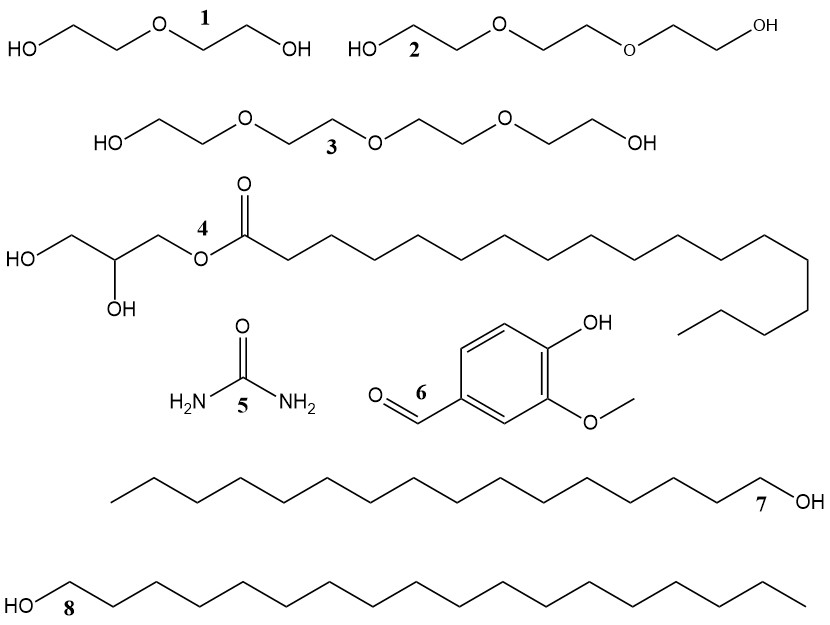


Figure S1D Molecular structure of glycol and other compounds (1 - diethylene glycol, 2 - triethylene glycol, 3 – tetraethylene glycol, 4 – 1-monostearin, 5 – urea, 6 – vanillin, 7 – hexadecanol, 8 – octadecanol


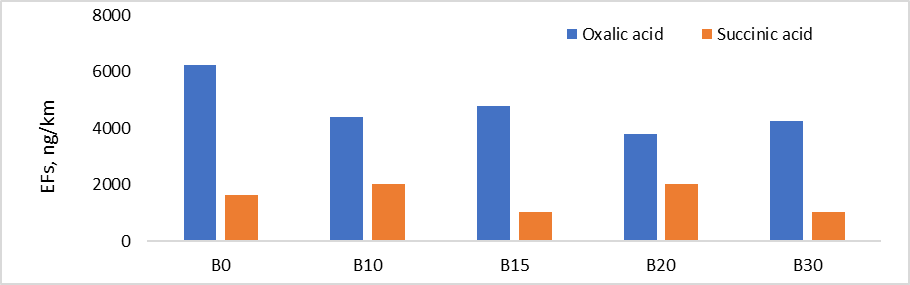

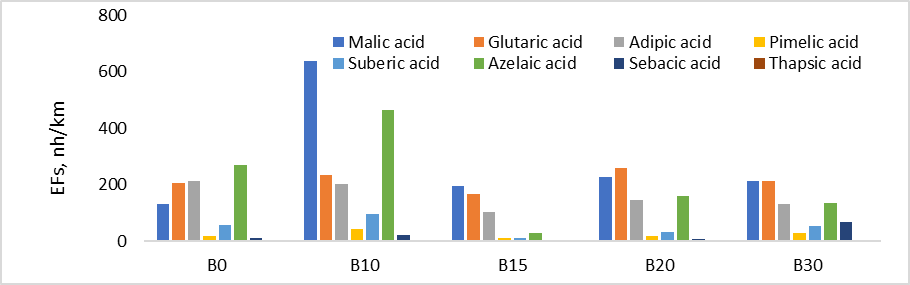


Fig S2 Emissions factors (sum of all particle size fractions) of dicarboxylic acids for different types of fuels. Note: the UF fraction of B15 is missing because its extract was lost.

Fig S3 Emissions factors (sum of all size fractions) of hydroxy acids for different types of fuels. Note: the UF fraction of B15 is missing because its extract was lost.


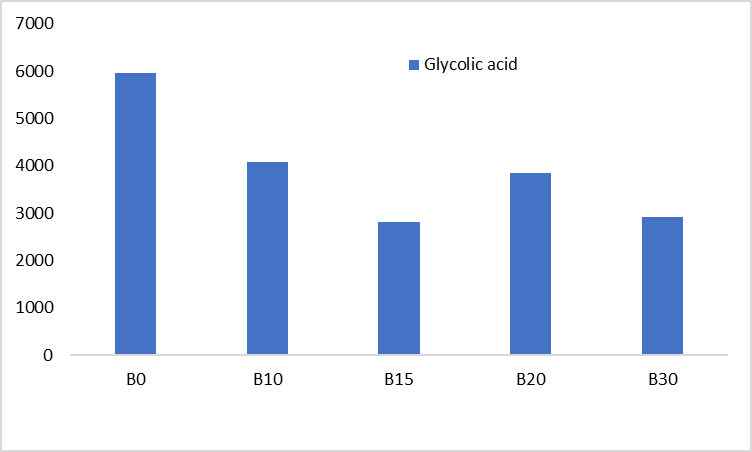

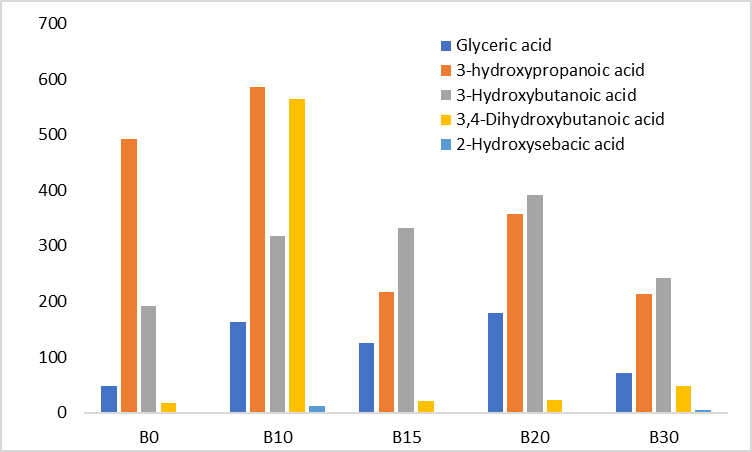

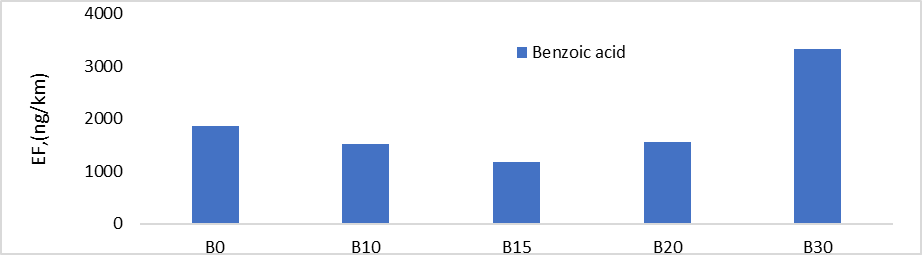

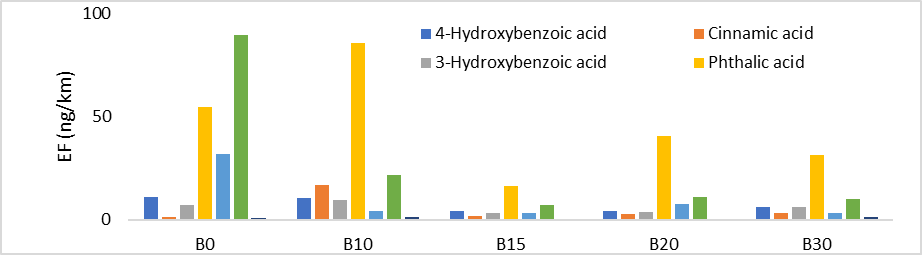


Fig S4 Emissions factors (sum of all size fractions) of aromatic acids for different types of fuels. Note: the UF fraction of B15 is missing because its extract was lost.

Fig S5 Emissions factors (sum of all size fractions) of alkanoic acids for different types of fuels. Note: the UF fraction of B15 is missing because its extract was lost.
